# Characteristics and outcome of spontaneous bacterial meningitis in patients with diabetes mellitus

**DOI:** 10.1186/s12879-020-05023-5

**Published:** 2020-04-20

**Authors:** Virginia Pomar, Natividad de Benito, Albert Mauri, Pere Coll, Mercè Gurguí, Pere Domingo

**Affiliations:** 1 Infectious Diseases Unit (Department of Internal Medicine), Hospital de la Santa Creu i Sant Pau – Institut d’Investigació Biomèdica Sant Pau. Universitat Autònoma de Barcelona, Barcelona, Spain; 2 Emergency Department, Hospital de la Santa Creu i Sant Pau, Barcelona, Spain; 3 Department of Clinical Microbiology, Hospital de la Santa Creu i Sant Pau. – Institut d’Investigació Biomèdica Sant Pau, Universitat Autònoma de Barcelona, Barcelona, Spain

**Keywords:** Spontaneous meningitis, Bacterial meningitis, Bacterial infection of the central nervous system, Diabetes mellitus

## Abstract

**Background:**

Studies on bacterial meningitis in diabetics patients versus non-diabetics are scarce. In patients with diabetes, bacterial meningitis may have a different presentation, etiology and course. We analyzed and compared the characteristics and outcome of spontaneous BM in adult patients with and without diabetes mellitus (DM).

**Methods:**

We performed a single-center, prospective observational cohort study, conducted between 1982 and 2017, in a tertiary university hospital in Barcelona (Spain). The primary outcome measure was in-hospital mortality.

**Results:**

We evaluated 715 episodes of bacterial meningitis; 106 patients (15%) had diabetes mellitus. Patients with diabetes were older (median 67 [IQR 17] vs 49 [IQR 40] years, *p* <  0.001) and more often had a Charlson comorbidity score of ≥3 (40% vs 15%, *p* <  0.001). Neck stiffness (56% vs 75%, p <  0.001), headache (41% vs 78%) *p* <  0.001), nausea and/or vomiting (32% vs 56% *p* < 0.001), and rash (12% vs 26%, *p* = 0.007) were less frequent in diabetics, whereas altered mental status was more common. *Streptococcus pneumoniae* and *Listeria meningitis* were the most common etiologic agents (24 and 18%, respectively). *Listeria* was more frequent (18% vs. 10%, *p* = 0.033), whereas meningococcal meningitis was less frequent (10% vs 32%, *p* < 0.001). Overall mortality was higher in patients with diabetes (26% vs 16%, *p* = 0.025) concerning non-diabetics.

**Conclusions:**

Patients with bacterial meningitis and diabetes mellitus are older, have more comorbidities, and higher mortality. *S. pneumoniae* and *L. monocytogenes* are the predominant pathogens, *Listeria* being more common, whereas *Neisseria meningitidis* is significantly less frequent than in non-diabetics.

## Background

Bacterial meningitis (BM) remains a significant cause of infection-related death worldwide [1, 2]. In industrialized countries, its estimated annual incidence is 4–6 cases per 100,000 adults, and *Streptococcus pneumoniae* followed by *Neisseria meningitidis* rank first among the causative agents [1–6]. The prevalence of BM mainly depends on the patient‘s age, geography, and season of the year. Lately, vaccines against meningeal pathogens have shown a meaningful impact, and will hopefully further modify this scenario shortly [7].

The International Diabetes Federation estimated that there were almost 400 million people living with diabetes mellitus (DM) around the world in 2016. Its prevalence is expected to increase to more than 590 million people by the year 2035 [8]. There is a positive association between diabetes and the development of several types of infection. Many aspects of immunity are altered in patients with DM, because hyperglycemia decreases neutrophil and monocyte function, including impaired chemotaxis, adherence, and phagocytosis. Therefore, patients with diabetes tend to be hospitalized for infections more frequently than non-diabetics [9–16].

The incidence of spontaneous BM in diabetic patients is unknown since most of the previous reports have combined spontaneous and post-neurosurgical meningitis, or diabetes has not been the focus of the study [9, 17–19]. A recent study describes community-acquired bacterial meningitis in patients with and without diabetes, but this study focused on community-acquired, culture-proven meningitis [20]. In our study, we included nosocomial infections and also unknown etiology BM. The underrepresentation of these patients in the Netherland cohort may lead to prevalence and mortality underestimation.

In previous studies we observed that DM increases among older patients, and that DM was the associated comorbidity in 13% of adults [4, 21].

Moreover, compared to non-diabetic patients, the presentation and course of common infections in diabetic patients may vary, leading to a possible delay in diagnosis and therapy. The causative organisms may also differ from those identified in the general population, depending on the glycemic control, local nosocomial trends, and specific vulnerabilities created by other associated underlying comorbidities, such as alcoholism and cancer or DM-related complications [18–20]. Diabetic patients seem to be at particularly high risk of infection with certain bacterial microorganisms, such as *Streptococcus agalactiae*, *Klebsiella spp*, *Salmonella enteritidis*, and *Staphylococcus aureus* [22–24].

Our study aims to compare BM characteristics and outcome in patients with and without DM for 36 years. Such a piece of knowledge may improve the medical management, prognosis, and mortality of these patients.

## Methods

### Setting and study population

We collected data from 1982 through 2017 at the Hospital de la Santa Creu i Sant Pau (Barcelona, Spain), a 540-bed tertiary university hospital serving an estimated population of 450,000 in a predominantly urban area. We included all consecutive adult patients (> 14 years) with spontaneous bacterial meningitis.

The characteristics of this cohort have been described elsewhere [4, 21, 25, 26], and also how we identified the cases. Data were collected during the hospitalization and the patient was monitored after discharge by one of the authors (PD or VP). Follow-up always included a neurological exam, neuropsychological testing, complement, and immunoglobulin levels, and audiometry.

The ethics committee approved the study and its subsequent amendments of the *Hospital de la Santa Creu i Sant Pau*. Verbal informed consent was obtained from all participants (patients, parents, or guardians) because of the years and the kind of the study. Our ethic committee accepts this situation.

### Diagnosis

The diagnosis of culture-proven BM was made on the basis of consistent clinical findings, a positive CSF culture. If CSF culture was negative, culture-proven BM could still be diagnosed with CSF neutrophilic pleocytosis (= > 100 neutrophils/cu mm), a positive CSF antigen test, or a positive blood culture. Meningococcal meningitis was diagnosed, in addition to those culture-proven, in patients with CSF gram-negative diplococci identified together with with a petechial or purpuric rash and a fulminant course [27].

Unknown etiology BM was diagnosed in patients with a compatible clinical picture together with neutrophilic pleocytosis and decreased CSF glucose (defined as CSF/blood glucose ratio < 0.40) or elevated CSF proteins (defined as > 0.5 g/l) [3, 6, 28].

Patients with a history of neurosurgical procedures or traumatic head/spinal injuries and cases of viral, fungal, or mycobacterial meningitis were excluded.

### Microbiology methods

Isolate identification and susceptibility tests performance have been described elsewhere [4, 21, 25, 26, 29–31].

### Definitions

Majority of the variables have been defined in other published works [4, 21, 25, 26].

The Charlson Comorbidity Index was used to assess **comorbidity** [32, 33]. Comorbid conditions were considered to be present if the patient had a confirmed diagnosis of one or more.

A diagnosis of **DM** was based on information provided by the patient or his/her family, recorded on medical charts or due to the presence of diagnostic criteria for DM at the time of admission (glycosylated hemoglobin [HbA1c] ≥ 6.5% or a fasting plasma glucose level ≥ 126 mg/dl [7 mmol/L] or the 2-h plasma glucose value after 75 g oral glucose tolerance test > 200 mg/dl [11,1 mmol/L] or in a patient with classic symptoms of hyperglycemia [polyuria, polydipsia or polyphagia] or hyperglycemic crisis, random plasma glucose ≥200 mg/dL [11.1 mmol/L]) [34]. We included type 1 and 2.

A **primary distant focus** of infection was considered when a patient had clinical symptoms and signs consistent with a focal infection distant from the central nervous system, and the same pathogenic bacterium was isolated from the primary focus of infection or a blood culture. Communication of the subarachnoid space with the skin, sinuses, or mucosal surfaces, and upper respiratory tract infection (frequent in meningococcal disease) were not considered distant foci of infection [24].

The **symptoms-admission interval** (SAI) was the interval, in hours, between onset of signs and symptoms of BM and admission to hospital was. When the onset of symptoms could not be precisely determined, we calculated the mean interval between the last time the patient was asymptomatic, observed by a household member, and the first time the patient was seen ill [35].

The **admission-therapy interval** (ATI) was the interval, in hours, between hospital admission and first dose of antibiotics for the treatment of meningitis.

Impaired mental status, seizures and focal neurological signs (motor, sensory or cranial nerve disturbances) detected on admission or subsequently were considered **neurological complications**. Coma was defined as a score of 6 or less on the Glasgow Coma Scale in the absence of sedation [36].

The development of septic shock, acute respiratory failure, acute renal failure, and/or consumption coagulopathy was considered a **systemic complication** if it was related to bacterial meningitis and was apparent on admission or shortly afterwards [24, 37].

**Adequate antibiotic treatment** was defined as the intravenous administration of any antimicrobial agents to which isolated bacteria were sensitive following susceptibility testing at local laboratories, which crossed the blood-brain barrier in adequate amounts, were administered in a dose recommended for acute BM treatment and commenced on the day of admission or before deterioration of neurological and systemic conditions in inpatients [24, 38, 39].

**Dexamethasone** therapy was only considered when its first dose of at least 10 mg/24 h was administered prior to or concomitant with the first antibiotic dose. Steroids administered after starting antibiotic therapy were not considered [40].

**Nosocomial meningitis** was defined as developing more than 48 h after admission or within one week of discharge [41].

**Inpatient mortality** was considered to be due to BM when meningitis was the underlying and immediate cause of death. BM-related death was defined and recorded if there were no other cause and if there were agreement between the reviewing physician and the underlying cause of death recorded on the medical records. We classified the causes of death in two categories: neurological causes (intractable seizures, brain herniation, cerebrovascular complications or coma) and systemic causes (septic shock, respiratory failure, disseminated intravascular coagulation or multiple organ failure) [37, 42]. Sepsis resulted from a host’s systemic inflammatory response syndrome (SIRS) to infection with 2 or more of temperature > 38 °C or < 36 °C, heart rate > 90, respiratory rate > 20/min or pCO2 < 32 mmHg or white blood cell count > 12,000/mm3 or < 4000/mm3 or > 10% immature bands. Septic shock was defined as sepsis induced hypotension persisting despite adequate fluid resuscitation. Disseminated intravascular coagulation is primarily a laboratory diagnosis, based on the combination of elevated fibrin-related markers (FRM), with decreased procoagulant factors and platelets.

**Sequelae** were defined as any disability, disorder, or injury demonstrated during hospital stay or upon discharge from hospital that was not present before the episode of BM and persisted at 6 months after discharge [42].

### Statistical analysis

Qualitative variables were expressed as absolute numbers and percentages, whereas quantitative variables were expressed as means and standard deviation (SD).

The comparison of continuous variables was performed through Student’s t-test or Mann-Whitney U test, whereas categorical data were compared by c2 test or Fisher’s exact test.

Independent predictive factors for mortality were explored through logistic regression analysis with adjustment for clinically relevant covariates was used, and the outcome shown sed as adjusted odds ratios. The proportion of the total variation of the outcome explained by a model was assessed through Nagelkerke’s R2.

All the tests were two-tailed. Significance was established by a *p*-value < 0.05. Statistical Product and Service Solutions (SPSS) software, version 19 (SPSS Inc., Chicago, IL) was used for all the statistical calculations.

## Results

During the 36-year study period we diagnosed 715 episodes of spontaneous acute bacterial meningitis. The median age were 55 years (IQR 38) and etiology was established in 583 cases (81.5%). *N. meningitidis* was the most common microorganism overall (28.7%), followed by *S. pneumoniae* (25.6%), *L. monocytogenes* (11.6%) and gram-negative bacilli other than *Haemophilus influenzae* (5.9%).

Of these 715 adults included in the study, 106 (15%) had diabetes mellitus: 45 (42%) diagnosed between 1982 and 1999, and 61 (58%) between 2000 and 2017 (*p* < 0.002).

### Characteristics of spontaneous bacterial meningitis (BM) in patients with diabetes mellitus

The demographic characteristics and clinical features of the population are summarized in Table 1. Patients with DM were older and have a Charlson comorbidity score of ≥3 more often than non-diabetics. When the Charlson score was recalculated excluding DM without and with end-organ failure, the comorbidity score for the two groups was similar (Charlson comorbidity index ≥3 was 18/106 [17%] in diabetic patients versus 89/609 [15%], *p* = 0.555).

**Table 1:** Demographics and clinical features of spontaneous bacterial meningitis episodes in diabetic patients

Characteristics	Diabetic Patients (*n* = 106)	Other Patients (*n* = 609)	*P* value
Male sex	53 (50)	298 (48.9)	0.916
Age – years, median (IQR) Recurrent meningitis	67 (17) 6 (5.7)	49 (40) 26 (4.3)	< 0.001 0.455
Comorbid conditions^a^	106 (100)	284 (46.6)	< 0.001
• Hypertension	19 (17.9)	61 (10)	0.028
• Cancer	17 (16)	89 (14.8)	0.541
• Chronic lung disease^b^	13 (8.2)	39 (6.4)	0.098
• Alcoholism	11 (7.2)	55 (9)	0.210
Charlson comorbidity index ≥3	42 (39.6)	90 (14.8)	< 0.001
Distant focus of infection	41 (38.7)	200 (32.8)	0.266
Route of acquisition			
• Hospital-acquired (vs. community-acquired)	4 (3.8)	28 (4.6)	1.000
Symptoms on presentation			
• Fever	100 (94.3)	579 (95.1)	0.849
• Altered mental status	83 (78.3)	383 (62.9)	0.009
• Neck stiffness	59 (55.7)	458 (75.2)	< 0.001
• Triad of fever, neck stiffness, and change in mental status	44 (41.5)	282 (46.3)	0.577
• Headache	44 (41.5)	473 (77.7)	< 0.001
• Nausea and/or vomiting	34 (32.1)	340 (55.8)	< 0.001
• Focal neurological deficits	25 (23.6)	100 (16.4)	0.095
• Coma	21 (19.8)	94 (15.4)	0.254
• Seizures	10 (9.4)	49 (8)	0.819
• Rash	13 (12.3)	160 (26.3)	0.007
• Systolic blood pressure – mm Hg (SD)	140 (32)	128 (30)	< 0.001
• Diastolic blood pressure – mm Hg (SD)	80 (19)	75 (20)	0.004
Interval symptoms-admission, hours (IQR)	25 (51.7)	24 (28)	0.184
Interval admission-therapy, hours (IQR) Prior antimicrobial therapy Cerebral computed tomography	4 (9) 28 (26.4) 45 (42.5)	3 (8) 185 (30.4) 192 (31.5)	0.821 0.647 0.039
White blood cell count, median (IQR)	15,025 (8525)	15,300 (10650)	0.704
Platelet count/mm3, median (IQR)	206,000 (252275)	189,500 (120750)	0.715

^a^Patients may have more than one co-morbid condition. ^b^Includes: chronic bronchitis, asthma, idiopathic pulmonary fibrosis, other pneumopathies. *CI* confidence interval. *IQR* interquartile range, *SD* standard deviation

Values are reported as no. / no. evaluated (%), unless otherwise noted

Hypertension and cancer were the most common underlying conditions in these patients.

The median blood glucose level in people with DM was 12.4 mmol/l (IQR 7.6), statistically higher than in non-diabetics: 7.4 mmol/l (IQR 2.9), *p* < 0.001.

A distant focus of infection was present in 41 of the 106 episodes (39%), with otitis or sinusitis (12 episodes, 11%), upper respiratory tract infection (7 episodes, 7%) and pneumonia (6 episodes, 6%) being the most frequently diagnosed distant foci.

Altered mental status was more frequent among patients with DM than among non-diabetics, whereas neck stiffness, headache, nausea, and rash were less frequent.

### Diagnosis and microbiology

Lumbar puncture was performed in all episodes, and the CSF showed at least one CSF finding suggestive of acute bacterial meningitis: increased protein levels in 103 cases (97%), a decreased CSF glucose/blood glucose ratio in 83 cases (78%), and pleocytosis with an elevated neutrophil count in 100 cases (94%). CSF cyto-biochemical findings did not differ significantly between the two groups of patients (Table 2). On the contrary, blood cultures were more frequently positive in diabetic patients (63% vs. 45%, *p* < 0.001) (Table 2).

**Table 2:** CSF findings, microbiologic features and etiology of bacterial meningitis in diabetic patients

Characteristics	Diabetic Patients (*n* = 106)	Other Patients (*n* = 609)	*P* value
CSF examination			
• White blood cell count, median (IQR)	960 (2672)	1008 (2670)	0.723
• Protein, g/l, median (IQR)	3.2 (6.4)	3.4 (4.75)	0.445
• CSF/plasma glucose ratio, median (IQR)	0.31 (0.36)	0.21 (0.36)	0.079
Positive CSF gram-stained smear	37 (34.9)	255 (41.9)	0.57
Positive CSF culture	81 (76.4)	429 (70.4)	0.39
Positive blood culture	67 (63.2)	273 (44.8)	< 0.001
Etiology			
• Meningococcal	11 (10.4)	194 (31.9)	< 0.001
• Pneumococcal	26 (24.5)	154 (25.3)	0.632
• Listeria and gram-positive bacilli^a^	19 (17.9)	64 (10.5)	0.033
• Gram-negative bacilli^b^	10 (9.4)	32 (5.3)	0.114
• *Haemophilus influenza*	3 (2.8)	16 (2.6)	0.753
• Other^c^	17 (16)	30 (4.9)	< 0.001
• Mixed^d^	0 (0)	4 (0.7)	1.000
• Unknown origin	16 (15.1)	115 (18.9)	0.415

Gram-positive bacilli^a^: *Listeria monocytogenes* (79) and *Bacillus* spp. (4). Gram-negative bacilli^b^: *Escherichia coli* (17), *Pseudomonas aeruginosa* (9), *Pseudomonas spp* (3), *Klebsiella spp* (2), *Proteus mirabilis* (2), *Serratia marcescens* (1), *Serratia spp* (2), *Enterobacter cloacae* (1), *Citrobacter freundii* (1), *Bacteroides melaninogenicus* (1), *Acinetobacter baumannii* (1), Fusobacterium (1). Other^c^: *S. agalactiae* (9), *Staphylococcus aureus* (8), *Enterococcus faecalis* (3), *S. pyogenes* (3), *S. viridans* (3), *Brucella spp* (2), *Neisseria subflava* (1), *Staphylococcus anginosus* (1), *Staphylococcus lugdunensis* (1), *Staphylococcus* spp. (19, Group G *Streptococcus* (1). Mixed meningitis^d^: *Bacteroides intermedius* + *Streptococcus viridans* (1), *E*.*cloacae* + *S.viridans* (1), *Peptostreptococcus spp* + anaerobic unidentified gram negative bacilli (1), and *Peptostreptococcus spp* + *Bacteroides spp* (1)

*CI* confidence interval, *IQR* interquartile range, *SD* standard deviation, *CSF* cerebrospinal fluid

Values are reported as no./no. evaluated (%), unless otherwise noted

The most common organisms were *S. pneumoniae* (24% in people with DM vs. 25% in non-diabetic patients, *p* = 0.632) followed by *L. monocytogenes* (18% vs. 10%, respectively, *p* < 0.033). There were only 11 (10%) cases of meningococcal meningitis, which was significantly more common in non-diabetic patients (194 episodes, 32%, *p* < 0.001). *S. agalactiae* was detected in 9 diabetic patients (8.5%) and in 6 non-diabetic patients (0.98%), with OR = 9.32 (2.88–32.42), *p* < 0.001. (Table 2). The etiologic spectrum of BM did not vary significantly between the two periods of the study except for listerial meningitis, which was more frequent in the second period: 3 (7%) vs. 16 (26%) cases (*p* = 0.01).

### Treatment

There was a high number of patients with DM having received out-of-hospital antibiotic therapy, although not statistically different from the rest of the population (Table 1).

Initial empirical antibiotic treatment was appropriate in 93 patients with diabetes (88%), being less frequent, although not statistically significant, than for non-diabetic patients (93%, *p* = 0.109) (Table 3). There was no difference between the 2 periods (37 [40%] vs. 56 [60%], *p* = 0.230). The most organisms not adequately covered by initial empiric therapy were *L.monocytogenes*, *Pseudomonas aeruginosa,* and *Staphylococcus aureus*.

**Table 3:** Evolving features and outcome of bacterial meningitis in diabetic patients

Characteristics	Diabetic Patients (*n* = 106)	Other Patients (*n* = 609)	*P* value
Neurological complications	31 (29.2)	123 (20.2)	0.210
• Coma	21 (19.8)	94 (15.4)	0.254
• Seizures • Focal neurological deficits • Cranial palsies	18 (17) 8 (7.5) 7 (6.6)	70 (11.5) 19 (3.1) 28 (4.6)	0.148 0.046 0.337
Systemic complications	48 (45.3)	184 (30.2)	0.024
• Acute respiratory failure	28 (26.4)	103 (16.9)	0.029
• Acute kidney failure	27 (25.5)	72 (11.8)	< 0.001
• Septic shock	19 (17.9)	88 (14.4)	0.376
• Disseminated intravascular coagulation • Rhabdomyolysis	11 (10.4) 6 (5.7)	47 (7.7) 22 (3.6)	0.677 0.286
Therapeutics			
• Adequate empiric antibiotic therapy	93 (87.7)	568 (93.3)	0.109
• Dexamethasone therapy • Vasoactive drugs • Mechanical ventilation • Dialysis	39 (36.8) 17 (16) 17 (16) 3 (2.8)	220 (36.1) 77 (12.6) 88 (14.4) 15 (2.5)	0.910 0.350 0.839 0.895
Outcome			
• Neurological sequelae	8 (7.5)	19 (3.1)	0.046
• In-hospital mortality	27 (25.5)	97 (15.9)	0.025

*CI* confidence interval, *IQR* interquartile range, *SD* standard deviation

Values are reported as no./no. evaluated (%), unless otherwise noted

The median duration of antibiotic treatment was 15 days (IQR 11).

There was no difference between the two groups in the administration of adjunctive steroids before or together with the first dose of antibiotic treatment (37% vs. 36%, *p* = 0.910).

### Outcome

Neurological complications were not different between patients with and without DM. However, the presence of focal neurological deficits after discharge (between survivors) was more frequent in diabetics (7.5% vs. 3.1%, *p* = 0.046) (Table 3).

Systemic complications were also more frequent in diabetics (45% vs. 30%, *p* = 0.024).

The overall mortality rate was significantly higher in patients with DM (25% vs. 16%, *p* = 0.025) (Table 3), being higher in the first study period (18 between 1982 and 1999 (66.7%) vs. 9 between 2000 and 2017 (33.3%), *p* = 0.006). The BM-attributable death in diabetic patients was 52%, and among non-diabetics was 63% (*p* = 0.72). The mean number of days until death did not differ between diabetic and non-diabetic patients (14 vs. 15, *p* = 0.857). Independent factors associated with an unfavorable outcome by multivariate analysis included: septic shock, coagulation disorder, age 65 or older, acute renal failure, the periods of the study, and gram-negative bacillary etiology. Diabetes was not associated with higher mortality (Table 4).

**Table 4:** Multivariate analysis for effect on unfavorable outcome

Variable	Odds Ratio	95% CI	*P* value
**Septic shock**	**8.896**	**4.272–18.527**	**<0.001**
**Coagulation disorder**	**4.108**	**1.585–10.646**	**0.004**
**Age (≥65 years)**	**2.311**	**1.352–3.950**	**0.002**
**Acute renal failure**	**2.238**	**1.206–4.152**	**0.011**
Impaired mental status	1.612	0.797–3.261	0.184
Charlson comorbidity index ≥3	1.594	0.902–2.818	0.109
Positive CSF culture	1.219	0.596–2.492	0.588
Positive blood culture	1.223	0.689–2.170	0.493
Triad of fever, neck stiffness, and change in mental status	0.801	0.443–1.450	0.464
Diabetes mellitus	0.762	0.398–1.460	0.413
White cell count in CSF > 1000/mm3	0.664	0.390–1.132	0.132
**Period time**	**0.552**	**0.317–0.962**	**0.036**
Adequate empiric antibiotic therapy	0.270	0.129–0.565	0.001
Etiology			
• *Neisseria meningitidis*	1.000 (reference)		
• *Streptococcus pneumoniae*	1.045	0.463–2.359	0.916
• *Listeria monocytogenes*	1.150	0.483–2.738	0.753
• **Gram-negative bacilli**	**2.893**	**1.043–8.027**	**0.041**

*CI* confidence interval. Nagelkerke’s R^2^ for the adjusted model = 0.446

## Discussion

Diabetes mellitus is steadily increasing in western societies, and its association with the development of several types of infection is well-known [4, 8, 11, 16, 19–21]. On the other hand, common infections in diabetic patients may be caused by unusual pathogens, and the presentation and course may be different from those without DM, which may delay the correct diagnosis and the adequate therapy [9–15, 17–19].

Previous studies, as well as our own, have shown an increased frequency of BM in patients with DM over the 36-year study period [16, 20], but in contrast to a study by van Veen KEB, et al. [20] the classic symptoms of meningitis were present less often than in non-diabetics. The reason could be the immunosuppression, but also to the fact that this group is older and with other underlying diseases, both factors being associated with atypical presentations [21]. Despite the complex immune system dysfunction associated with diabetes, our patients displayed increased white cell blood counts.

There are significant differences in the regional distribution of the etiological agents of BM [43], and the available vaccines have led to further changes in the etiologic spectrum of bacterial meningitis. In our investigation, *S. pneumoniae* was the most frequent pathogen in patients with DM, while *L. monocytogenes* ranked second, and it was much more frequent than in patients without diabetes. The main risk factors for listerial meningitis are age (newborns an elderly), pregnancy, cancers and immunosupression [5, 21, 44, 45]. Given the complex immunosuppression caused by DM, this finding was expectable. Some other uncommon pathogens have been described [18, 19]. In our study *N.meningitidis* is uncommon in diabetic patients concerning other patients, so diabetes mellitus does not seem to be a predisposing condition for meningococcal meningitis. However, it is logical to see that diabetes can be present in older adults who have meningococcal meningitis, and most likely, this is a product of age together with the age-dependent trend in the incidence of meningococcal disease.

On the other hand, *S. agalactiae* was detected more frequently in diabetic patients. The rate of *S. agalactiae* infections in adults is thought to be increasing, with an estimated twofold to fourfold increase in its incidence in non-pregnant adults reported in the past two decades [24]. Though infrequent, *S. agalactiae* has emerged as an outstanding etiologic agent of bacterial meningitis in adults with severe co-morbid conditions [24].

Moreover, we found that diabetic patients had more co-morbid conditions, and this may have had an impact on the etiology and prognosis of bacterial meningitis.

As other previous studies have shown [17, 46], the CSF parameters were diagnostic for bacterial meningitis and did not differ between the two groups; therefore, these parameters were still helpful for the diagnosis of meningitis.

Cerebral computed tomography (CT) was more frequently performed among diabetic patients in our study, maybe due to the greater availability of such a tool in the second half of the study. However, most probably, the attending physicians tend to consider diabetic patients as immunocompromised. Fortunately, this was not associated with an increased ATI, which carries a worse prognosis [3, 4, 39, 40]. Nevertheless, this interval needs to be reduced if we want to improve the outcome of our patients. Current international guidelines have proposed the “red flags” for identifying patients that need a cranial imaging before lumbar puncture: focal neurological deficits, new-onset seizures, severely altered mental status, and severely immunocompromised state [39]. Following these guidelines, most of the cerebral computed tomography performed in our patients was unnecessary.

In diabetic patients, if the CSF Gram stain is negative, or it not seems a pneumococcal meningitis, empiric antibiotic coverage should always be directed at *L. monocytogenes* [3, 5]. In contrast with other studies [19], diabetes patients were not less likely to be treated with adjunctive dexamethasone therapy.

Furthermore, DM influences the outcome of specific infections, such as bacteremia and mortality following pneumococcal pneumonia [12, 22]. Compared to non-diabetic patients with BM [19, 20], we found a higher mortality rate among patients with diabetes, and 52% of mortality was attributable to meningitis. The probable reasons were age, the frequent presence of comorbidities, altered mental status on admission, and the development of complications such as renal failure. The mortality improved between the two study periods, most likely due to the adjunctive dexamethasone therapy, as guidelines recommend [2, 40, 47, 48], and the higher rate of adequate empiric antibiotic therapy. However, we cannot exclude that improvements in the management of critical patients, and diabetes and its complications may have played a role.

Given the fact that *S. pneumoniae* is the leading cause of BM in diabetics and that pneumococcal infections are potentially vaccine-preventable, it is worth recommending such vaccination in people with DM.

Our study has inherent limitations. It is based on a single hospital, and therefore the results may not apply to other geographical areas or populations. Secondly, the number of diabetic patients was relatively small, although the present study is one of the largest to date. Thirdly, the long study period may integrate changes in the medical procedures or in the antimicrobial resistance, changes in the modern comorbidities therapies, and the introduction of vaccines in the population. Fourthly, although there can be an overlap in the cyto-biochemical profile between bacterial and mycobacterial or viral meningitis and meningitis without proven pathogen, the increasing availability of diagnostic tools for these etiologies led to the refinement of the diagnosis of meningitis of unknown origin, and we honestly feel that the possibility of misdiagnosis is it is minimal. Finally, we have no data about the BM characteristic by type of diabetes due to the years of the study.

## Conclusions

In summary, bacterial meningitis remains a highly lethal complication in diabetic patients. Our report shows that the symptoms and the etiology of meningitis in diabetic patients differ from those in non-diabetics. A high index of suspicion, and the reduction of the time between admission and adequate therapy, which should include coverage against *L. monocytogenes*, could aid in the outcome of diabetic patients.

## Abbreviations

ATI: Admission-therapy interval
BM: Bacterial meningitis
CI: Confidence interval
CLSI: Clinical Laboratory Standards Institute
CSF: Cerebrospinal fluid
CT: Computed tomography
DM: Diabetes mellitus
HbA1c: Glycosylated hemoglobin
HIV: Human immunodeficiency virus
IQR: Interquartile range
MICs: Minimal inhibitory concentration
SAI: Symptoms-admission interval
SD: Standard deviation
SPSS: Statistical Product and Service Solutions
